# Evaluation of Methods to Minimize Pain in Newborns during Capillary Blood Sampling for Screening: A Randomized Clinical Trial

**DOI:** 10.3390/ijerph19020870

**Published:** 2022-01-13

**Authors:** Magdalena Napiórkowska-Orkisz, Aleksandra Gutysz-Wojnicka, Mariola Tanajewska, Iwona Sadowska-Krawczenko

**Affiliations:** 1 Department of Midwifery, School of Health Sciences, Collegium Medicum, University of Warmia and Mazury in Olsztyn, Żołnierska 14c Street, 10-561 Olsztyn, Poland; 2 Department of Nursing, School of Health Sciences, Collegium Medicum, University of Warmia and Mazury in Olsztyn, Żołnierska 14c Street, 10-561 Olsztyn, Poland; a.gut.wojnicka@uwm.edu.pl; 3 Department of Neonatology and Intensive Therapy of a Newborn, Provincial Specialist Hospital in Olsztyn, Żołnierska 18 Street, 10-561 Olsztyn, Poland; tanajewskamariola@gmail.com; 4 Department of Neonatology, Ludwik Rydygier Collegium Medicum in Bydgoszcz, Nicolaus Copernicus University in Torun, ul. Ujejskiego 75, 85-168 Bydgoszcz, Poland; iwonasadowska@cm.umk.pl

**Keywords:** neonatal pain, pain assessment, pain minimization, non-pharmacological analgesia, screening

## Abstract

Aim: The aim of the study was to assess the severity of pain experienced by a newborn during a heel puncture for screening using the Newborn Pain Scale (NIPS), measure the heart rate and compare the effectiveness of non-pharmacological methods of pain control. Design: Randomized clinical trial. No experimental factors. The test was performed during routine screening. Surroundings: Provincial Specialist Hospital in Olsztyn. Patients/Participants: Pain was assessed in 90 full-term newborns. The newborns were rooming in with their mothers in the hospital. Interventions: Newborns were divided into three groups. Three different methods of pain relief were used: breastfeeding, 20% glucose administered orally and non-nutritional sucking. Main Outcome Measures: The primary pain outcome was measured using the NIPS and the secondary pain outcome measures (heart rate, oxygen saturation) were measured using a pulse oximeter. Results: During capillary blood sampling from the heel, most newborns, *n* = 56 (62.2%), experienced no pain or mild discomfort, severe pain occurred in *n* = 23 (25.6%) and moderate pain occurred in *n* = 11 (12.2%). No significant statistical differences were found between the degree of pain intensity and the intervention used to minimize the pain *p* = 0.24. Statistically significant relationships were demonstrated between heart rate variability and the degree of pain intensity (*p* = 0.01). There were no statistically significant differences between the newborn’s pain intensity and the mother’s opinion on the effectiveness of breastfeeding in minimizing pain. Conclusions: This study did not answer the question of which pain management method used during the heel prick was statistically more effective in reducing pain. However, the results indicate that each of the non-pharmacological interventions (breastfeeding, oral glucose dosing and non-nutritive sucking) applied during heel puncture resulted in effective pain management in most of the newborns enrolled in the study. The relationship between heart rate variability and the severity of pain was confirmed. Mothers of newborns in the breastfeeding group were satisfied with the pain relief methods used in the child and the opportunity to console their newborn during painful procedures in a technologically invasive environment.

## 1. Introduction

The newborn screening program is the only procedure that enables early detection, diagnosis and treatment of several dozen congenital diseases that are life-threatening, disturb development and lead to irreversible neurological changes and severe intellectual disability. Currently, in the Republic of Poland, as part of the 2019–2022 edition of the program, newborns are screened for the entire population, which includes 30 congenital diseases. The screening test is performed within 48 h of childbirth in the first days of the life of every child born in Poland. The exclusion criterion from participation in the screening program is the lack of acceptance of the screening test expressed in writing by the mother/legal guardian of the child [1,2]. Across the globe, the newborn screening process is evolving with the understanding of health conditions, while the availability of diagnostic tests and treatment options are improving [3]. Blood sampling is the leading cause of pain in hospitalized newborns. The heel prick seems to be one of the most common painful methods of blood sampling in the infant population. The collection of capillary blood for a screening test by a puncture of the medial lateral side of the heel is associated with pain experienced by newborns [1,2]. This technique is popular because it enables the collection of a very small volume of blood (0.2–0.5 mL). The use of this technique requires proper preparation of the child’s foot, adherence to the principles of infection control measures before and during the procedure, and the use of non-pharmacological methods of pain relief before starting the procedure [4]. The Polish Neonatal Society recommends using non-pharmacological methods of pain relief for heel puncture in newborns [5]. Aside from humanitarian and ethical concerns, inadequate treatment of pain may have long-lasting physiological and neurodevelopmental consequences, including increased susceptibility to chronic pain syndromes and a heightened sensitivity to subsequent painful stimuli which may persist throughout childhood [6]. Neonatal pain control is therefore an important part of newborn care. The pain assessment tool used for neonates should be multidimensional, including measurements for both physiological and behavioral indicators of pain. There are numerous scales that have been validated in both term and preterm neonatal populations, including the Premature Infant Pain Profile (PIPP), the Neonatal Infant Pain Scale (NIPS), CRIES, the COMFORT scale, Neonatal Pain, the Agitation and Sedation Scale (N-PASS) and the Face, Legs, Activity, Cry, Consolability scale (FLACC) [2].

Many studies have been performed to find the best non-pharmacological way to reduce pain in infants, which mainly include skin-to-skin contact, Kangaroo Mother Care (KMC), tucking by parents, glucose solutions such as dextrose, non-nutritive sucking in term neonates, and breastfeeding [7,8,9,10,11,12,13,14,15,16]. It is assumed that breastfeeding reduces pain sensations through three different mechanisms: endorphin release due to the sweet taste, skin contact and cradling during breastfeeding and the sucking reflex. The authors of the Cochrane review reported (2017) that skin-to-skin care, in which newborns wearing only a diaper are held next to their mother’s bare chest, has many benefits, including improved breast milk production, breastfeeding duration, parent satisfaction, sleep organization and a longer duration of quiet sleep [17]. Vu-Ngoc et al. (2019) reported that non-nutritive sucking is a safe and effective pain-relief method during the heel prick procedure in term neonates, which can routinely be used as a pain-relief method in infants [10]. Mosayebi et al. (2014) stated that KMC before and during heel lancing is a natural, easy to use and cost-effective method to decrease pain in premature neonates [8]. Kassab et al. found that facilitated tucking by parents (FTP) and dextrose water D10W were effective methods for managing and decreasing pain among full-term neonates following the heel lance procedure. The results also showed that using dextrose water D10W was more effective than using FTP [9]. Soltani et al. (2018) conducted a double-blind, controlled, randomized clinical trial to compare the efficacy of four methods of relieving infant pain: breastfeeding, oral 25% dextrose, kangaroo mother care method (KMCM) and local anesthetic agents, such as EMLA cream (lidocaine 2.5% and prilocaine 2.5%) following heel-prick sampling in term newborns. The authors reported that the most effective method of reducing pain in infants undergoing painful procedures was proven to be breastfeeding [12]. 

To date, there is no conclusion concerning the best method to reduce pain sensations in healthy full-term newborns and the pain scales used to evaluate the pain. 

## 2. Objective

The aim of this study was (1) to assess the intensity of pain experienced by a newborn child during a heel prick for a screening test using the Neonatal Infant Pain Scale (NIPS) and measurements of physiological parameters (heart rate, oxygen saturation); (2) to compare the effectiveness of non-pharmacological methods of pain control applied during a painful procedure, and (3) to identify mothers’ opinions about breastfeeding quality during painful procedures experienced by a newborn.

## 3. Research Ethics

This study was approved by the Ethics Committee of Nicolaus Copernicus University in Torun, Collegium Medicum in Bydgoszczy, Poland, Approval No KB 206/2015 of 23 February 2015. One of the parents/legal guardians of each newborn provided written consent for participation in the clinical trial, which included the name and surname of the examined person, number of the patient’s medical history and the date and signature of the parents. 

## 4. Design

This was a pragmatic randomized clinical trial (RCT) conducted to compare the efficiency of non-pharmacological methods to reduce heel-prick pain among full-term, healthy newborns at a provincial hospital in Olsztyn, Poland. Three methods of pain reduction were tested: breastfeeding vs. oral glucose vs. non-nutritive sucking. This pragmatic trial was conducted in the provincial hospital from 1 March to 15 May 2015. The number of newborns included in the study was determined by the duration of the RCT. The pain stimulation of a heel prick was applied in relation to the routine newborn screening process. The omission of a control group that was not treated with a pain minimizing agent in the study was due to humanitarian reasons.

## 5. Population

Healthy infants participated in the study. The inclusion criteria were gestational age: 38–42 weeks of pregnancy, birth weight ≥ 2500 g, Apgar score in the 5th minute > 7 points, over 48 h of age. Excluded from the trial were infants diagnosed with malformations or a remarkable, unusual finding in their physical examinations.

## 6. Blinding and Allocation

This study was performed without blinding due to the specificity of the pain assessment tool and pain reduction methods used. The painful intervention, pain assessment tool and analgesia methods were performed concurrently over time. The infants were allocated randomly using envelopes that contained the three groups, i.e., I, II, III. Newborns were randomly assigned to one of three groups that differed in pain management methods. Group I consisted of newborns who were attached to the mother’s breast during the painful procedure. Group II included those given 2–3 mL of 20% glucose orally. Group III sucked a pacifier during the study. A pool of tickets was prepared in which there were 30 tickets qualifying for one of the three randomization groups. 

## 7. Study Instrument

The Neonatal Infant Pain Scale (NIPS) questionnaire was used to evaluate the perceived pain level in infants. It was developed by Lawrence et al. [11] and is recommended for newborns and infants with a gestational age of 28–38 weeks for the assessment of procedural pain. NIPS includes indicators such as respiratory patterns, crying, appearance of the face, upper and lower limb movements and alertness. The NIPS scoring system is as follows: crying (0: no cry, 1: whimper, 2: vigorous); facial expression (0: relaxed, 1: grimace); breathing pattern (0: relaxed, 1: change in breathing pattern); arm and leg movements (0: relaxed, 1: flexed/extended); state of arousal (0: sleeping/awake, 1: fussy), and the total pain score ranges between 0–7. Each observed behavior is scored, and those scores are then summed. The higher the score, the higher the pain level is assumed. Pain assessment data were recorded using an Excel spreadsheet. When interpreting the scale, it was assumed that the points obtained on the scale mean: 0–3 no pain to mild discomfort, 4–5 moderate pain, and 6–7 severe pain. Both the validity and reliability of this questionnaire were approved by Lawrence et al. [11]. Alpha coefficients and inter-item correlation were calculated to determine the internal consistency of the Polish version of the NIPS and its subscale. The total Cronbach coefficient for the Polish version of the NIPS was 0.8697, and the average correlation between items was 0.5584. 

## 8. Organization

The clinical trial took place in the maternity ward of a provincial hospital. After obtaining permission from the parent and confirming the eligibility of the newborn, the participating newborns and their mothers were invited to a consultation room for the heel prick procedure. Demographic information regarding the newborns was collected from their parents. After the mother drew a lottery ticket with a number, the newborn was assigned to the appropriate randomization group. Each included neonate was connected to an electronic monitor to check physiological data (HR, oxygen saturation) and then underwent the procedure of a heel prick for a screening test using the pain relief method assigned to the group. Efforts were made to ensure that the amount of blood collected in each newborn was the same. The blood soaked to the other side of the tissue and the blood discs were a maximum of 1 cm. The first drop was always discarded. Automatic lancets that penetrate the skin to a depth of no more than 2.4 mm were used for heel puncture. The heel was punctured at the sole of the heel in the lateral area. The heels were not heated. Each time before blood sampling, attention was paid to the condition of the skin and sampling from previous puncture sites was avoided (e.g., after collecting capillary blood for glucose level testing or blood gas analysis).

## 9. Procedure

The primary pain outcome was measured using the NIPS and the secondary pain outcome measures (physiological pain responses) were checked using a pulse oximeter. Additionally, mothers of children from the first randomization group (breastfeeding group) after completing the heel prick procedure were asked to answer four closed questions regarding their opinion on the effectiveness of the method used to minimize pain. The questions were related to the weaning of the newborn from breastfeeding during the heel puncture, success in grasping it again, feelings related to the power of sucking the breast and the mother’s opinion on the effectiveness of the method used to minimize the procedural pain. During the collection of capillary blood from the heel, the reactions of all newborns included in the study were assessed on the behavioral pain scale NIPS (Neonatal Infant Pain Scale) by one of the authors (an academic teacher) and recorded in the data collection sheet. The physiological parameters and any adverse reaction were observed and recorded by a nursing student assigned to the individual mother and her child during clinical practice conducted in this ward. Mothers were also invited to report any important events which took place during the procedure. The heel puncture was performed by a full-time midwife employed in the ward according to the hospital policy regarding newborn screening tests.

## 10. Trial

**Figure 1:**
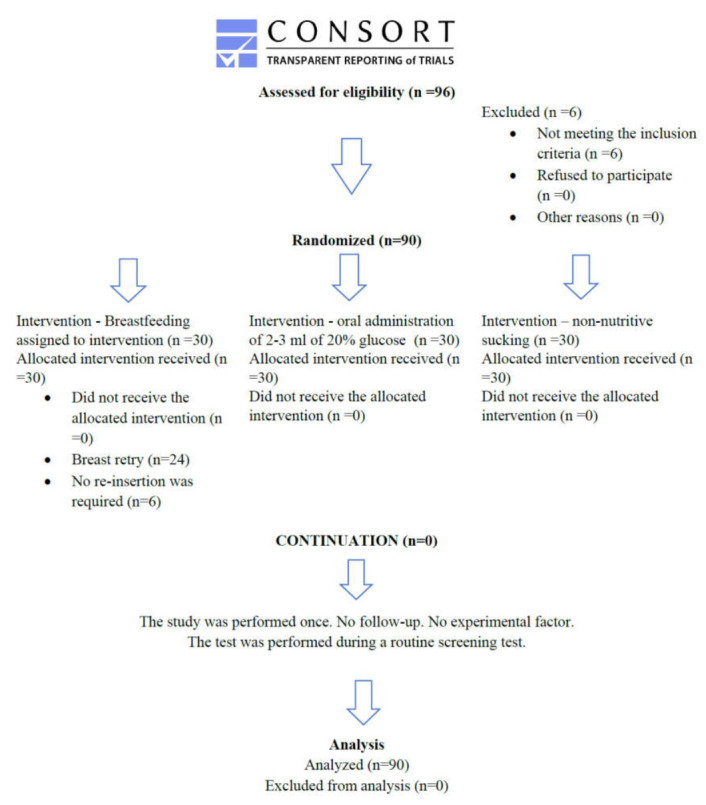
CONSORT diagram.

Pain was assessed in 90 newborns staying with their mothers in the rooming-in system at the Provincial Specialist Hospital in Olsztyn from 1 March to 15 May 2015. Randomization was used in the study. Six newborns were excluded from the study because they did not meet the inclusion criteria [18] (Figure 1 CONSORT diagram).

The mothers of the newborns assigned to the first group (breastfeeding) were instructed in the correct technique of latching the newborn to the breast before starting the capillary blood collection procedure. In this case, the time of blood collection depended on the planned feeding time. The mother was informed about what reactions from the newborn could be expected after the heel puncture. Before the procedure, it was assessed whether the breastfeeding was proceeding properly. In the case of difficulties, every effort has been made to solve the lactation problem. The position of the newborn during breastfeeding was agreed with the mother. The feeding position needed to be accepted by the mother, child and midwife performing the blood collection (it should allow easy access to the newborn’s heel and not overload the midwife’s movement apparatus). After completing the heel prick procedure, the mother was asked for her opinion on the effectiveness of the method used to minimize pain, and all answers were recorded in the data collection sheet. Newborns included in the second group were orally given 2–3 mL of 20% glucose prepared in a syringe. A few drops of glucose were applied to the front of the tongue 1–3 s prior to the puncture of the newborn’s heel by another midwife, and the administration was continued during the procedure. The same volume of glucose was given for each newborn. In the case of the third group (non-nutrition sucking), the heel puncture took place when the infant started sucking on the pacifier. Prior to the procedure, the mother was instructed on how to use the pacifier and observe if, in response to pain, the newborn was pushing the teat with his tongue. In such a case, the mother was instructed to give the teat for a second time. 

## 11. Analysis

Statistica ver. 10 (StatSoft Polska, Kraków, Poland) was used in this study. An analysis of the significance of differences in the assessment of the severity of pain and the analyzed variables, which did not meet the necessary assumptions for the ANOVA test, was performed using the Kruskal–Wallis test when comparing several groups of variables, and the Mann–Whitney U test when comparing two independent samples. Spearman’s rank correlation coefficient was used to assess the occurrence of the relationship between individual variables and the strength of this relationship. The strength of the correlation is based on the following scale: rxy = 0 variables are not correlated; 0 < rxy < 0.1 low correlation; 0.1 ≤ rxy < 0.3 weak correlation; 0.3 ≤ rxy < 0.5 average correlation; 0.5 ≤ rxy < 0.7 high correlation; 0.7 ≤ rxy < 0.9 very high correlation; 0.9 ≤ rxy < 1 correlation almost complete. The significance level of *p* < 0.05 was adopted in all calculations.

## 12. Results

### Assessment of Pain Using the NIPS Scale

During capillary blood sampling from the heel, the pain experiences of newborns, according to the NIPS scale, were as follows: most newborns, *n* = 56 (62.2%), experienced no pain or mild discomfort, severe pain occurred in *n* = 23 (25.6%), and moderate pain occurred in *n* = 11 (12.2%) (Table 1). 

When analyzing the severity of pain in individual groups, it was observed that in the breastfeeding group, *n* = 21 (70%) newborns did not feel pain or felt a slight discomfort (1 to 3 points on the NIPS). In the second group (20% glucose given orally), *n* = 19 (63.3%) felt no pain or slight discomfort, and in group three (non-nutritional sucking), *n* = 16 (53.3%) newborns experienced no pain or felt slight discomfort. Further results are presented in Table 1. No significant statistical differences were found between the degree of pain intensity and the intervention used to minimize the pain *p* = 0.24 (Table 2).

## 13. The Opinion of Mothers on Minimizing Pain Sensations during Breastfeeding

The mothers of the newborns assigned to the first intervention group (breastfeeding) were asked to answer four closed-ended questions. 

(1) Did the infant wean off the breast during the heel-prick procedure?

As many as *n* = 24 (80%) newborns in response to the pain stimulus weaned from the mother’s breast, while 6 (20%) newborns did not release their breasts. In the group of newborns who weaned from the mother’s breast during blood collection, the mean pain intensity was 3.2 (SD = 2.5). In the group of newborns who did not wean the mother’s breast, the mean pain intensity was 0.5 (SD = 0.5). Among the six newborns who did not wean, the pain was rated at the maximum 1 point on the NIPS scale. The test revealed a statistically significant relationship between the weaning of the breastfeeding and the severity of pain at the significance level of *p* = 0.003 (Table 3).

(2) Was it possible to reattach the newborn to the breast?

Most newborns *n* = 18 (75%) who weaned in response to the pain stimulus had no major problems with reattachment. On the other hand, *n* = 5 (21%) of the respondents did not grasp the mother’s breast again. For one newborn (4%), it was quite difficult, but it worked. A statistically significant relationship was found between the pain assessment and the success of reattachment to the breast. A lower intensity of pain was much more common in newborns who managed to grasp the breast again. In the group of newborns who were easily attached to the breast during blood collection, the mean pain intensity was 1.9 (SD = 1.4). In the group of newborns who failed to reattach to the breast, the mean pain intensity was 7.0. The six newborns who did not wean during the study were excluded from the analysis. The test revealed a statistically significant relationship between the re-grasping of the breast and the pain intensity level at the significance level of *p* = 0.01 (Table 4).

(3) Did you feel a difference in the power of your baby sucking on your breast?

Most mothers *n* = 17 (68%) did not notice a significant change in the newborn’s sucking power during the procedure; *n* = 7 (21%) experienced a change in sucking after the next attachment. There was a statistically significant relationship between the pain rating and the difference in infant sucking power compared to previous feedings. It was observed that newborns whose mothers did not report a difference in sucking power significantly more often did not experience the pain associated with the painful procedure. Among the 17 newborns whose mothers did not experience a significant change in suckling power, the mean pain intensity was 1.2 (SD = 1.0). The mean pain intensity of the seven infants who sucked the breast more forcefully after re-grasping was 3.3, (SD = 2.0). The test revealed a statistically significant relationship between the newborn’s suckling power and the degree of pain intensity at the significance level of *p* = 0.02 (Table 5).

(4) Do you think that conducting the test during breastfeeding minimized the pain sensation in your child?

In the study, 24 (80%) mothers considered breastfeeding as an effective method of minimizing pain, *n* = 4 (13%) gave a negative answer, and *n* = 2 (7%) mothers believed that the use of the above-mentioned method had no analgesic effect. There were no statistically significant differences between the newborn’s pain assessment and the mother’s opinion on the effectiveness of breastfeeding in minimizing pain.

In the group of 24 mothers who considered the method effective, the average pain perception in the newborns was 1.8 (SD = 1.8). In the group of four mothers who did not confirm the effectiveness of the method used, the average perception of pain by the newborns was 6.8 (SD = 0.5). All four newborns showed severe pain. Among the two mothers who believed that the method used was irrelevant in minimizing pain, the average neonatal pain perception was 4.0, (SD = 4.2) (Table 6). 

## 14. Heart Rate Variability

The greatest variability in heart rate occurred in the group of newborns sucking a pacifier, and the lowest was after oral administration of 20% glucose. Analyzing the relationship between heart rate variability and the degree of pain intensity, statistically significant relationships were demonstrated at the significance level of *p* = 0.01. The lowest heart rate variability occurred in the group of newborns who did not feel pain and scored between 0 and 3 points on the NIPS scale. On the other hand, the highest variability occurred in the case of experiencing severe pain. A correlation was found between pain rating according to the NIPS scale and heart rate variability (Table 7).

The mean difference between peak and baseline heart rate was greatest in the third group of newborns (non-nutritive sucking) and was 29.2 (SD = 17.7). In the first group (breastfeeding), it was 23.1 (SD = 15.2), and in the second group (20% glucose administrated), the mean heart rate variability was 22.3 (SD = 15.2) (Table 8).

## 15. Discussion

Heel puncture is one of the most painful procedures performed in newborns. This study was conducted to compare the efficiency of breastfeeding vs. oral glucose administration and vs. non-nutritive sucking in reducing heel-prick pain among full-term, healthy newborns. The assessment of the intensity of pain experienced by a newborn was conducted using the Neonatal Infant Pain Scale (NIPS) and analyzing the changes in physiological parameters such as heart rate and oxygen saturation. To all newborns undergoing the painful procedure, one of the pain-reducing interventions was applied. This study did not result in answering the question of which applied pain relief intervention is statistically more significant in reducing pain during the heel-prick procedure in newborns. However, the results demonstrate that each of the non-pharmacological interventions (breastfeeding, oral glucose administration and non-nutritive sucking) applied during the heel prick resulted in successful pain management in the majority of newborns included in the trial. The data suggest that breastfeeding is the most effective pain relief intervention, followed by oral glucose administration and non-nutritive sucking. However, these findings were not statistically confirmed. These findings are supported by the work of Soltani et al., who reported that among the different methods of pain management in newborns, the most effective method of lowering perceived pain was proven to be breastfeeding, followed by dextrose administration [19]. On the other hand, Kassab et al. reported that using 2 mL of dextrose water D10% was an effective method of providing analgesic effects on full-term neonates [9]. However, it should be noted that Kassab et al. did not compare the effectiveness of dextrose application vs. breastfeeding in pain management in newborns in their study [9]. Moreover, non-nutritive sucking, another method used in the current study, was proven by Vu-Ngoc et al. to be a safe and effective pain-relief method during the heel prick procedure in term neonates [10]. The current study confirmed the relationship between heart rate variability and the degree of pain intensity. The highest variability occurred in the case of experiencing severe pain. The mean difference between peak and baseline heart rate was greatest in the non-nutritive sucking group and was the lowest in the 20% glucose intervention group. This finding may suggest that non-nutritive sucking is a less effective method of pain relief in newborns. However, these findings were not confirmed statistically. In many countries, including Poland, there is a deficiency in the knowledge and practice of neonatal pain management. The need for the education of health professionals on neonatal pain management and national guidelines for pain management was reported by Panek et al. [20]. The non-pharmaceutical pain-reducing interventions used in this trial were safe and easy to apply. No side effects were observed. Mothers of the newborns assigned to the breastfeeding group were satisfied with the pain relief methods applied to the child and confirmed the effectiveness of breastfeeding as a method of pain reduction. Furthermore, breastfeeding provided mothers with an opportunity to comfort their neonates during painful procedures in a technologically invasive environment. 

## 16. Conclusions

Blood sampling is a painful procedure routinely performed in newborns. Therefore, efforts must be made to relieve this pain. The literature indicates that easy-to-use and cost-effective non-pharmacological methods to decrease pain in newborns have proven effects on reducing procedural pain in newborns. Although this study did not provide an answer to the question of which non-pharmacological method is the most effective in reducing pain in newborns, new data were added to the body of knowledge regarding pain management among neonates. It appears that among methods such as breastfeeding, administrating glucose and non-nutritive sucking, the most effective method of lowering perceived pain in newborns is breastfeeding. Breastfeeding was proven to be widely accepted by mothers who wanted to be involved in procedures to reduce the pain of newborns. 

## 17. Implications for Practice

The current study highlights the need for more research to determine the most effective method of pain management in newborns. Since neonatal centers have no guidelines for the treatment of pain in newborns, natural, easy-to-use, and cost-effective methods to decrease pain should be introduced to clinical practice. The findings of this study support the use of methods such as breastfeeding, administering glucose and non-nutritive sucking in reducing pain, although statistically significant differences were not found between them. Special attention should be paid to breastfeeding as it is not only an effective pain-relieving method but also a method that encourages parents to participate in the provision of care for their babies.

## Figures and Tables

**Table 1:** Assessment of pain in newborns by intervention group.

Pain Scale (Points)	Groups of Newborns			
	Total (*n* = 90)	Breastfeeding (*n* = 30)	20% Glucose Administered (*n* = 30)	Non-Nutritional Sucking (*n* = 30)
No pain (1–3 pts)	62.2% (*n* = 56)	70.0% (*n* = 21)	63.3% (*n* = 19)	53.3% (*n* = 16)
Moderate pain (4–5 pts)	12.2% (*n* = 11)	10.0% (*n* = 3)	16.7% (*n* = 5)	10.0% (*n* = 3)
Strong pain (6–7 pts)	25.6% (*n* = 23)	20.0% (*n* = 6)	20.0% (*n* = 6)	36.7% (*n* = 11)

**Table 2:** Statistical correlation between pain assessment and intervention groups.

Parameter	Total (*n* = 90)	Groups of Newborns			*p*
		Breastfeeding	20% Glucose Administrated	Non-Nutritional Sucking	
Pain assessment using NIPS	3.1 ± 2.5 ^(1)^ 2.5 ^(2)^ 0–7 ^(3)^	2.6 ± 2.5 ^(1)^ 2.02 ^(2)^ 0.0–7.0 ^(3)^	3.1 ± 2.2 ^(1)^ 3.0 ^(2)^ 0.0–7.0 ^(3)^	3.7 ± 2.6 ^(1)^ 3.0 ^(2)^ 0.0–7.0 ^(3)^	0.24 *

* statistically significant differences *p* < 0.05, Kruskal–Wallis test ^(1)^ mean ± standard deviation, ^(2)^ median, ^(3)^ range.

**Table 3:** The relationship between pain assessment and the answer to question no. 1.

Question	Answer	Number of Respondents Answering the Question (*n* = 30)	Pain Assessment	*p*
Did the newborn baby detach from the feeding breast?	Yes	24	3.2 ± 2.5 ^(1)^ 2.0 ^(2)^ 0.0–7.0 ^(3)^	0.003 *
	No	6	0.5 ± 0.5 ^(1)^ 0.5 ^(2)^ 0.0–1 ^(3)^	

* statistically significant differences *p* < 0.05, Kruskal–Wallis test ^(1)^ mean ± standard deviation, ^(2)^ median, ^(3)^ range.

**Table 4:** Relationship between pain assessment and the answer to question no. 2.

Question	Answer	Number of Respondents Answering the Question (*n* = 30)	Pain Assessment	*p*
Was it possible to reattach the newborn to the breast during blood collection?	Yes	18	1.9 ±1.4 ^(1)^ 2.0 ^(2)^ 0.0–5.0 ^(3)^	0.01 *
	No	5	7.0 ±0.0 ^(1)^ 7.0 ^(2)^ 7.0–7.0 ^(3)^	
	It was difficult	1	6.0 ± 0.0 ^(1)^ 6.0 ^(2)^ 6.0–6.0 ^(3)^	

* statistically significant differences *p* < 0.05, Kruskal–Wallis test ^(1)^ mean ± standard deviation, ^(2)^ median, ^(3)^ range.

**Table 5:** Relationship between pain assessment and the answer to question no. 3.

Question	Answer	Number of Respondents Answering the Question (*n* = 30)	Pain Assessment	*p*
Do you feel a difference in your baby’s suckling force compared to previous feedings? Did the baby suck the breast with more force, and if so, at what point?	I did not feel a distinct change	17	1.2 ± 1.0 ^(1)^ 1.0 ^(2)^ 0.0–3.0 ^(3)^	0.02 *
	At the moment of latching	1	1.0 ± 0.0 ^(1)^ 1.0 ^(2)^ 1.0–1.0 ^(3)^	
	After another latch on to the breast	7	3.3 ± 2.0 ^(1)^ 4.0 ^(2)^ 1.0–6.0 ^(3)^	

* statistically significant differences *p* < 0.05, Kruskal–Wallis test ^(1)^ mean ± standard deviation, ^(2)^ median, ^(3)^ range.

**Table 6:** The relationship between pain assessment and the answer to question no. 4.

Question	Answer	Number of Respondents Answering the Question (*n* = 30)	Pain Assessment	*p*
Do you think that conducting the test during breastfeeding minimized the pain sensations in your baby?	Yes	24	1.8 ± 1.8 ^(1)^ 1.0 ^(2)^ 0.0–7.0 ^(3)^	0.44 *
	No	4	6.8 ± 0.5 ^(1)^ 7.0 ^(2)^ 6.0–7.0 ^(3)^	
	Irrelevant	2	4.0 ± 4.2 ^(1)^ 4.0 ^(2)^ 1.0–7.0 ^(3)^	

* statistically significant differences *p* < 0.05, Kruskal–Wallis test ^(1)^ mean ± standard deviation, ^(2)^ median, ^(3)^ range.

**Table 7:** The relationship between heart rate variability and pain assessment using NIPS.

Pain Assessment NIPS	The Difference between Peak and Initial Heart Rate	*p*
0–3 points—no pain	17.5 ± 11.7 ^(1)^ 17.0 ^(2)^ 0.0–49.0 ^(3)^	0.01 *
4–5 points—moderate pain	29.5 ± 13.7 ^(1)^ 28.0 ^(2)^ 11.0–49.0 ^(3)^	
6–7 points—severe pain	40.6 ± 15.1 ^(1)^ 36.0 ^(2)^ 15.0–70.0 ^(3)^	

* statistically significant differences *p* < 0.05, Kruskal–Wallis test ^(1)^ mean ± standard deviation, ^(2)^ median, ^(3)^ range.

**Table 8:** The difference between the peak and the initial heart action in the groups of newborns.

Groups	Mean Difference between Peak and Baseline Heart Rate
Breastfeeding	23.1 ± 15.2
20% glucose administered	22.3 ± 15.2
Non-nutritive sucking	29.2 ± 17.7

mean ± standard deviation.

## Data Availability

The data presented in this study are available on request from the corresponding author.
